# A Framework for Integration of Machine Vision with IoT Sensing

**DOI:** 10.3390/s25237237

**Published:** 2025-11-27

**Authors:** Gift Nwatuzie, Hassan Peyravi

**Affiliations:** Department of Computer Science, Kent State University, Kent, OH 44240, USA; gnwatuzi@kent.edu

**Keywords:** machine vision, IoT sensing, sensor integration, multimodal fusion, edge–cloud computing, deep learning, automated monitoring

## Abstract

Automated monitoring systems increasingly leverage diverse sensing sources, yet a disconnect often persists between machine vision and IoT sensor pipelines. While IoT sensors provide reliable point measurements and cameras offer rich spatial context, their independent operation limits coherent environmental interpretation. Existing multimodal fusion frameworks frequently lack tight synchronization and efficient cross-modal learning. This paper introduces a unified edge–cloud framework that deeply integrates cameras as active sensing nodes within an IoT network. Our approach features tight time synchronization between visual and IoT data streams and employs cross-modal knowledge distillation to enable efficient model training on resource-constrained edge devices. The system leverages a multi-task learning setup with dynamically adjusted loss weighting, combining architectures like EfficientNet, Vision Transformers, and U-Net derivatives. Validation on environmental monitoring tasks, including classification, segmentation, and anomaly detection, demonstrates the framework’s robustness. Experiments deployed on compact edge hardware (Jetson Nano, Coral TPU) achieved 94.8% classification accuracy and 87.6% segmentation quality (mIoU), and they also sustained sub-second inference latency. The results confirm that the proposed synchronized, knowledge-driven fusion yields a more adaptive, context-aware, and deployment-ready sensing solution, significantly advancing the practical integration of machine vision within IoT ecosystems.

## 1. Introduction

Recent advances in sensing hardware, embedded computation, and intelligent data processing have created new opportunities for building autonomous monitoring systems. And yet, while this work has made great strides, unifying heterogeneous data sources into a coherent sensing workflow is still a key challenge. Conventional Internet of Things (IoT) nodes are suitable for high-frequency and scalar measurements—temperature, humidity, soil conditions or any additional environmental indicators—but their capabilities are limited to single-point signals with limited spatial context. In contrast, contemporary machine vision systems offer extensive visual information and detailed scene structure and are usually utilized as standalone subsystems that do not interface with wide-scale IoT architectures. This separation creates a fragmentation of data streams and hinders monitoring systems from providing a universal view of complex conditions. To fill this gap, this paper proposes a systematic framework that integrates machine vision and IoT sensors in a single, end-to-end monitoring architecture. Instead of treating visual and scalar measurements as independent modalities, the framework captures, processes, and fuses them into a cohesive framework that underlies context-aware reasoning and adaptive control. We leverage the visual information provided by imaging systems and the temporal accuracy offered by IoT networks to develop a more comprehensive and interpretable description of the field environment.

### 1.1. Machine Vision and IoT Sensing Integration Challenges

While each sensing technology has advanced at an accelerated pace, the interplay between machine vision and IoT networks is still not optimized. Environmental sensor arrays are a commonly used approach for accurately measuring temporal time and local trends [1], and their single-parameter architecture limits the way the system can reason about spatial patterns or visual cues. By contrast, deep learning-based machine vision models (e.g., Convolutional Neural Networks (CNNs) and Vision Transformers (ViTs)) effectively capture semantic structure, object appearance, and contextual features in images [2,3,4]. But these vision pipelines tend to exist in isolation, and many of these streams generate information streams that cannot interface with the environmental data generated by IoT sensors. Without coordinated processing, we do not have a network connection in place that enables the system to connect visual events to the scene environment. In the absence of a fusion mechanism, for example, a visual anomaly cannot be immediately associated with a temperature or humidity fluctuation, and it cannot be associated with the original atmospheric value. Prior research has demonstrated the advantages of combining data types [5], but scalable and algorithmically designed architecture, which bridges vision and IoT sensing all along the process pipeline, is still lacking. A comprehensive framework is needed to enable cross-modal reinforcement, enhance interpretability, and improve the robustness of automated monitoring systems.

### 1.2. Research Gaps and Proposed Framework

Current monitoring approaches tend to prioritize one modality over the other; IoT-centered systems emphasize energy efficiency and temporal measures, while vision-centered systems focus on retrieving semantic richness from images [6]. This split has led to a distinct research hole: the lack of a structured, end-to-end framework that could support heavily coordinated convergence between vision and IoT data for co-operative, context-sensitive intelligence. Although most existing systems generate fusion at the application layer [7], our work proposes a structured pipeline composed of well-defined architectural components to integrate low-level synchronization, cross-modal representation learning, and edge–cloud co-inferencing. To remedy this issue, we present an integrated architecture for the integration of machine vision and IoT sensing in a single model. The framework expands the idea beyond parallel data collection with joint inference, multimodal representation learning, and adaptive response mechanisms that are suitable for applications in the field. The major contributions of this work are summarized as follows:

- Unified Integration Architecture: We develop a comprehensive architecture that outlines the data flow synchronization and functional parts needed at scale for visual and IoT sensing streams to be integrated, from the sensor level upwards, and allows temporal syncing for joint inference and integration.
- Joint Multimodal Inference: We propose learning techniques that are able to directly infuse inference on fused data, using environmental conditions for enhanced visual interpretation and vice versa. This results in a more robust and accurate context for the whole system.
- Cross-Modal Knowledge Transfer: The framework has a mechanism to transfer learned representations between vision and IoT models, where the features from one modality are transferred to the other, strengthening the process and making learning regularized into the next one to improve generalization.
- Adaptive Edge–Cloud Coordination: We design a closed-loop operating strategy where real-time inference is computed at the edge, while the cloud resources maintain fine-tuning that allows periodic optimization of the model in response to changing field contexts.

The remainder of this paper is structured as follows: Section 2 reviews prior work on multimodal sensing and integration methodologies. Section 3 presents the proposed architecture and describes its core components. Section 4 provides experimental evaluation across multiple tasks. Section 5 discusses the broader implications, limitations, and potential extensions of the framework, and Section 6 concludes the study.

## 2. Related Works

Most of the development of multimodal monitoring systems has been taken along three different paths: scalar sensing networks, imaging-based monitoring, and recently, AI-driven vision analytics. However, these paradigms are still developing in isolation with a significant evolution of each paradigm to inform the development of environmental and agricultural monitoring. This separation constrains the field’s capability to establish such coordinated designs that can combine the spatial richness of visual data with the temporal precision of IoT measurements. In this section, we summarize the main pillars of work, identifying the critical necessity for an integrated sensing paradigm that bridges machine vision to IoT-driven data acquisition.

### 2.1. Scalar Sensing Technologies for Monitoring Systems

Scalar sensors continue to be cornerstones in most IoT monitoring projects and make continuous measurements of parameters such as temperature, humidity and soil moisture [6]. Since they are cheap and reliable, they have become an accepted tool in the environmental and agricultural fields. However, the fact that these sensors are confined to specific locations limits their spatial coverage in all spatial areas, as only a single node can monitor the local position of the same node, despite the fact that the environmental conditions may differ drastically over distance [8]. Dense sensor placement is needed to achieve wider coverage. However, dense sensor placement leads to higher costs, system operation maintenance difficulty, and increased costs associated with network maintenance. Lastly, scalar sensors cannot detect structural or visual cues such as discoloration, surface texture changes, or morphological patterns which are often used to identify environmental stress or abnormal behavior [5]. Although excellent in temporal accuracy and numerical accuracy, these tools are not interpretative in order to allow for deep understanding of the situation. This limitation demonstrates the importance of employing complementary sensing modalities for the capture of spatial and contextual information [9].

### 2.2. Technologies for Machine Vision and Imaging

Machine vision approaches are used due to the spatial constraints of the scalar sensing. The use of ground-based cameras, aerial platforms and multispectral or hyperspectral imaging systems provides detailed visual information at different scales [10]. For instance, UAVs provide widespread coverage and are used to perform plant investigations, detect anomalies, and aid with resource acquisition tasks. However, vision-led approaches operate with limitations. UAVs should follow airspace regulations, are weather-sensitive, and face computational load and flight endurance challenges. On the ground, imaging devices are stable but exist only in very narrow monitoring locations that often do not integrate with larger sensing systems in a direct manner. Therefore, because visual and scalar data often reside in separate lines of communication, unified analysis or collective decision support becomes rare.

### 2.3. AI for Multimodal Monitoring

Artificial intelligence has greatly advanced vision and IoT-based monitoring systems. Deep learning architectures, such as CNNs (Convolutional Neural Networks), ViTs (Vision Transformers), and hybrids, have displayed good performances for classification, detection, and segmentation tasks [2,4,11]. CNNs can identify local patterns, for instance, while transformer-based models can classify long-range dependencies and contextual relationships [12]. But for all that they can offer, the vast majority of AI-driven solutions are largely tailored to a single modality. They are typically trained on controlled data with low environmental variations, and they perhaps struggle to generalize across different forms of operational domains [5]. The challenge of deployment is also limited at the edge because of computational constraints for complex models to be run in an efficient manner. In addition, the interoperability of visual and scalar data streams is seldom considered in mainstream solutions, although both approaches may provide stronger and context-dependent monitoring [1,13]. This case highlights the demand for adaptable, scalable hybrid modalities architectures capable of leveraging the synergy between modalities.

### 2.4. Multimodal Fusion Frameworks

More recently, architectures integrating visual data with heterogeneous sensing modalities are attracting more attention. For example, ref. [7] explore a smart traffic management system combining video analytics and IoT-based road sensors. They provide strong evidence of the benefit of combining visual and scalar observations, but they utilize a late fusion scheme for modality integration, where each modality operates independently, before being merged at the decision layer. This is a limitation of synchronized feature interactions and of low-level cross-modal alignment. Edge–cloud collaboration systems constitute another branch of this field. Sathupadi et al. [14] provide an edge–cloud synergy framework intended for the real-time processing of industrial sensor network data. The system is efficient in distributing computation and minimizing latency, but its main domain is scalar sensor streams, and visual input and joint representations are not integrated between modalities. More generally, recent surveys such as that of Mondal et al. [15] underscore the increasing interest in multimodal event detection and provide an overview of the common data fusion strategies adopted in all domains. Beyond these dedicated environmental sensors, there is growing interest in using the communication infrastructure itself as a sensing modality. The field of Integrated Sensing and Communications (ISAC), for instance, investigates how wireless channel data can be used for perception. Ref. [16]’s work on ISAC imaging for 6G using ray tracing is a key example. While our work fuses data from dedicated cameras and IoT sensors, this illustrates the expanding universe of data sources available for environmental monitoring. Nevertheless, most of the schemes mentioned in the literature work at the application layer, and they tend to regard each modality as an independent resource of evidence. Such schemes fail to account for the necessity for fine temporal synchronization, shared feature spaces, or the inter-modal transfer of knowledge. Overall, the existing work confirms the good effects of multimodal sensing, but still less effort has been made in realizing very deep temporal fusion over several visual and IoT data streams. In the absence of a common architecture that allows for the synchronization of data acquisition and joint representation learning and adaptive edge–cloud co-ordination, there have been fragmented systems missing both the full complementarities of vision and IoT sensing. This void justifies the proposed integrated framework described in this work. The introduced framework fills a gap by incorporating vision and IoT sensing within a common edge–cloud context to provide complementary contributions to environmental interpretation, model learning, and decision support. As shown in Table 1, the framework enables the fused processing of data, cross-modal transfer of representation and adaptive feedback for the feedback systems adapted to the field characteristics. In contrast to previous work, which treats visual and scalar sensing as distinct or loosely associated operations, our design prioritizes deep integration throughout the pipeline to allow for scalable, context-aware and self-improving monitoring across many applications.

## 3. Methodology

**Figure 1:**
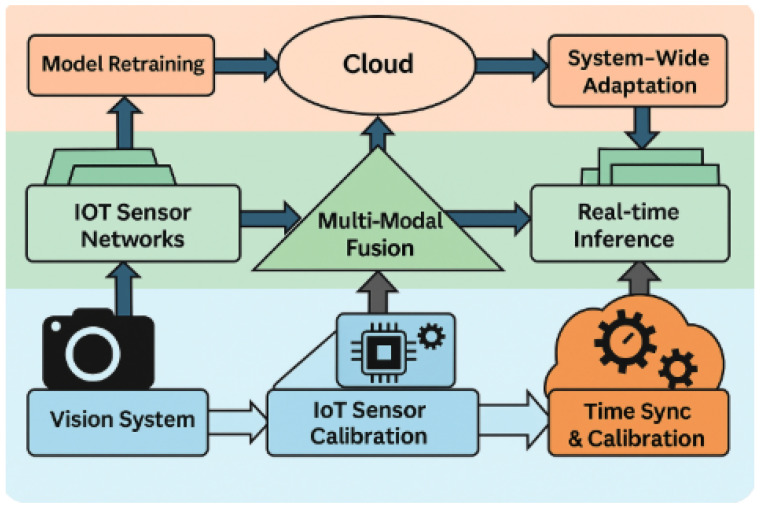
Integrated machine vision–IoT architecture showing synchronized data capture, multimodal fusion, edge inference, and cloud coordination.

This section introduces the unified framework designed to combine machine vision and IoT sensing within a coordinated monitoring pipeline. The architecture establishes a continuous flow of information from heterogeneous data acquisition to multi-task deep learning inference and distributed edge–cloud computation. As illustrated in Figure 1, the system is organized around three tightly connected layers that ensure consistent operation even under varying network conditions and environmental dynamics.

### 3.1. System Architecture Design

The proposed architecture follows a three-layer organization intended to support scalable, low-latency multimodal processing. The sensing layer forms the foundation of the system and includes fixed and mobile RGB cameras (12–16 MP); Sony IMX Series Sensors (Sony Semiconductor Solutions Corp., Tokyo, Japan), supported by synchronized environmental sensor modules. together with distributed IoT nodes measuring environmental variables such as temperature, humidity, and other scalar conditions. To ensure consistent temporal alignment across all sensing devices, the cameras and IoT nodes are synchronized using a lightweight IEEE 1588 Precision Time Protocol (PTP) service. This mechanism assigns a unified timestamp to each capture event, allowing visual frames and sensor packets to be matched even in cases where network delays occur. A small buffer at the edge device automatically discards packets that exceed an allowable drift threshold (typically 30–50 ms), preventing delayed sensor readings from misaligning with the corresponding image ROIs. This design ensures that downstream models receive tightly synchronized multimodal inputs.

The edge fusion layer serves as the primary computation stage for real-time inference. Implemented on embedded devices such as the NVIDIA Jetson Nano and Google Coral TPU, this layer performs feature extraction, compression, and cross-modal fusion under strict latency and power constraints. Several optimization strategies are employed, including structured pruning, quantization-aware training, and lightweight knowledge distillation, collectively reducing computation by up to 70% relative to uncompressed baselines. These adjustments preserve predictive accuracy while enabling deployment in energy-constrained environments. Temporal alignment from the sensing layer is preserved here so that the edge device can conduct joint inference using synchronized visual and scalar features.

The cloud intelligence layer manages global learning, long-horizon analytics, and system coordination. High-capacity models such as large-scale transformer architectures are trained and updated in this layer, using fused multimodal data aggregated over longer intervals. The cloud serves as a supervisory controller by pushing updated backbone weights to the edge at scheduled intervals (typically every 5–10 inference epochs or when performance drift is detected). This structure creates a digital replica of the monitored environment, allowing the cloud to refine anomaly detection, perform global trend analysis, and maintain system stability over time. Elastic computation ensures that the cloud scales according to data volume and model complexity while maintaining real-time feedback to the edge layer. The cloud-side model refinement employs a cosine embedding loss as the matching objective to align the feature representations from the vision and sensor modalities in a shared latent space.

### 3.2. Multimodal Data Preprocessing Pipeline

**Figure 2:**
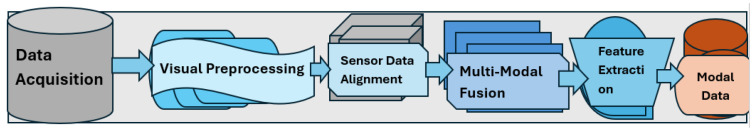
Preprocessing pipeline illustrating synchronized visual and sensor data operations prior to multimodal fusion.

The preprocessing pipeline shown in Figure 2 prepares heterogeneous data for unified analysis. Visual inputs are first normalized using color-constancy correction to reduce illumination variance, which is followed by a hybrid noise-reduction stage that applies median filtering for impulse noise and Gaussian smoothing for high-frequency artifacts. Essential edges and fine textures are retained to support downstream feature extraction. Region-of-interest (ROI) extraction is performed using YOLOv5 [18]. Since the sensing layer ensures synchronized timestamps, ROIs inherit the same temporal markers as their paired IoT measurements. When network-induced delays cause certain sensor packets to arrive late, the associated ROIs are either aligned using interpolation or excluded based on a drift threshold to prevent supervising the model with misaligned labels. To prevent misalignment between visual and sensor inputs, any image–sensor pair whose timestamps differ by more than a drift threshold τ is automatically discarded or temporally realigned before fusion.

Equation (1) shows the fusion of normalized visual and scalar features:

(1) $$Z=\mathrm{Integration}\left(f_{\mathrm{vision}}\left(I\right),f_{\mathrm{sensor}}\left(S\right)\right)=W_{v}f_{\mathrm{vision}}\left(I\right),⊕W_{s}f_{\mathrm{sensor}}\left(S\right)+b.$$

*Z* signifies the unified feature embedding formed from merging visual and sensor branch outputs. $W_{v}f_{\mathrm{vision}}\left(I\right)$ represents the feature vector obtained from the image stream, and $W_{s}f_{\mathrm{sensor}}\left(S\right)$ captures the value of the contributions from environmental sensor inputs. The bias *b* describes the affine transformation generated in fusion. The operation function ⊕ specifies the distinct technique that combined the two modalities, like concatenation, weighted addition, or a gated mixing solution. The notation Integration(·) is also used as this fusion process as it is not a mathematical integral but represents the mapping that creates a shared representation space of the two sets of features.

### 3.3. Multimodal AI Models and Integration Strategy

**Figure 3:**
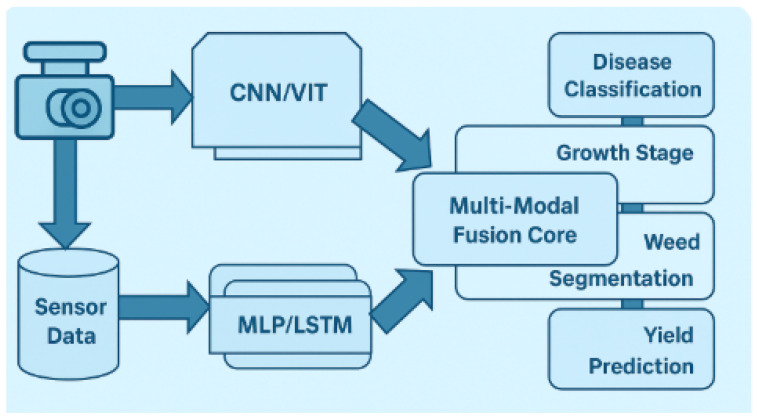
Fusion strategy illustrating independent feature extraction from visual and sensor streams prior to joint multimodal inference.

The integration strategy is based on a two-stream structure which allows for the separate processing of visual and scalar data and the integration of the visual and scalar data for joint inference (see Figure 3):

(2) $$Z=W_{v}\;\cdot \;f_{\mathrm{vision}}\left(I\right)⊕W_{s}\;\cdot \;f_{\mathrm{sensor}}\left(S\right)+b.$$

Equation (2) describes how the fused representation *Z* is formed by combining the processed outputs of the vision and sensor branches. $f_{\mathrm{vision}}\left(I\right)$ and $f_{\mathrm{sensor}}\left(S\right)$ represent the feature vectors obtained from image and sensor inputs, respectively. The matrices $W_{v}$ and $W_{s}$ apply learned transformations that adjust each feature set before they are merged. The bias term *b* completes the affine mapping. The operator ⊕ indicates the specific fusion rule used to bring the two modalities together. Further details on the motivation and structure of this fusion operator are previously explained in Equation (1).

To handle different learning requirements, several model families are incorporated. EfficientNet-B0 [19] provides robust visual feature extraction and is adapted with feature-wise modulation conditioned on sensor signals. For cross-modal knowledge transfer, the pretrained convolutional backbone (blocks 1–7) of EfficientNet-B0 is used as a fixed feature extractor. The final classification head is replaced, and the extracted features are then modulated by the sensor data embeddings before being passed to the task-specific layers. ViT-Tiny [2] models temporal dynamics by capturing long-range dependencies between image features and environmental trends. For pixel-level interpretation, U-Net with a ResNet-34 encoder [3,20] is used, where convolutional activations are modulated according to sensor context to improve segmentation consistency.

Joint optimization is formulated as shown below:

(3) $$L_{\mathrm{total}}=\sum_{T\in T}\lambda_{T}L_{T}\left(\theta \right)+\lambda_{1}{∥\theta ∥}_{2}^{2}+\lambda_{2}{∥\theta ∥}_{1}$$

where $\lambda_{T}$ is dynamically adjusted for each task during training. Smaller $\lambda_{T}$ values are assigned when gradient magnitudes grow excessively, following a simplified GradNorm-style balancing rule; this stabilizes multi-task optimization while reducing competition between tasks. Specifically, the weight for task *T* at training step *k* is updated as shown below:

(4) $$\lambda_{T}\left(k\right)=\lambda_{T}\left(k-1\right)\;\cdot \;{\left(\frac{∥\nabla L_{T}{∥}_{2}^{2}}{E_{T}[∥\nabla L_{T}{∥}_{2}^{2}]}\right)}^{\;\alpha }$$

where α is a damping hyperparameter that ensures tasks with disproportionately large gradients are down-weighted to maintain stable multi-task optimization.

Classification tasks use label smoothing to prevent overconfident predictions:

(5) $$\begin{array}{cc} y_{c}^{\mathrm{smooth}} & =\left(1-\epsilon \right)y_{c}+\frac{\epsilon }{C} \end{array}$$

(6) $$\begin{array}{cc} L_{\mathrm{cls}} & =-\sum_{c=1}^{C}\left[\left(1-\epsilon \right)y_{c}+\frac{\epsilon }{C}\right]\log\left({\hat{p}}_{c}\right)+\alpha \sum_{c=1}^{C}{\hat{p}}_{c}\log\left({\hat{p}}_{c}\right) \end{array}$$

where ϵ is the smoothing parameter and α is the entropy regularization weight.

For segmentation, the model uses a hybrid loss that combines Dice similarity and cross-entropy terms:

(7) $$L_{\mathrm{seg}}=\gamma \;\left(1-\frac{2|P\cap G|}{\left|P|+|G\right|}\right)+\left(1-\gamma \right)\;\left[-\frac{1}{N}\sum_{i=1}^{N}\sum_{c=1}^{C}y_{i,c}\log\left({\hat{p}}_{i,c}\right)\right]$$

This composite formulation improves spatial consistency and class balance, which is particularly beneficial for heterogeneous field imagery.

For regression estimation tasks, a robust Huber-style function is applied to handle outliers effectively:

(8) $$L_{\mathrm{reg}}=\frac{1}{N}\sum_{i=1}^{N}\left\{\begin{array}{cc} \frac{1}{2}{\left(y_{i}-{\hat{y}}_{i}\right)}^{2}, & \mathrm{if}\;|y_{i}-{\hat{y}}_{i}|\leq \delta ,\\ \delta |y_{i}-{\hat{y}}_{i}|-\frac{1}{2}\delta^{2}, & \mathrm{otherwise}. \end{array}\right.$$

This piecewise design ensures quadratic behavior for small residuals and a linear penalty for large deviations, improving robustness to noise and imperfect labels.

Classification tasks use label smoothing (Equation (6)) to prevent overconfident predictions, segmentation uses a Dice–cross-entropy hybrid loss (Equation (7)), and regression tasks rely on a robust Huber formulation (Equation (8)) to reduce sensitivity to outliers.

### 3.4. Optimization Framework and Training Methodology

**Figure 4:**
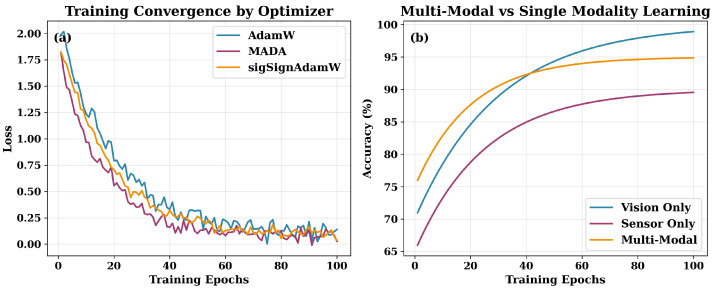
Convergence behavior showing (**a**) loss trajectories per optimizer and (**b**) accuracy gains due to multimodal integration.

The multimodal framework is trained in PyTorch2.0 with Automatic Mixed Precision (AMP), which decreases physical memory use and accelerates computations without degrading numerical robustness. To suit the heterogeneous and multi-task characteristics of the architecture, different optimization strategies were tested. Table 2 summarizes the performances of the optimizers experimented on and points out their unique respective strengths for different parts of the pipeline. AdamW [21] is selected as the standard optimizer for the visual backbone, which is primarily attributed to the decoupled weight decay and stable, monotonic convergence when extracting features. An adaptive momentum version known as an MADA is adopted to facilitate cross-modal alignment and thus to minimize the time error for the changing gradients between modalities by regulating the momentum term with an adaptive coefficient, promoting the convergence of the modality at the gradient level. The sigSignAdamW variant further enhances robustness for sensor-based tasks with the addition of sign-based variance normalization that supports robustness in contexts with gradient quality deterioration as a result of sensor noise. Figure 4 visualizes the optimization dynamics and indicates convergence paths and multimodal integration, which leads to accuracy in both cases improving every time.

The gradient update procedure begins by computing the composite gradient defined in Equation (9), which incorporates task-specific losses and both regularization terms:

(9) $$g_{t}=\nabla_{\theta_{t}}L_{\mathrm{total}}\left(\theta_{t}\right)+2\lambda_{1}\theta_{t}+\lambda_{2}\mathrm{sign}\left(\theta_{t}\right).$$

Momentum terms are then updated according to Equations (10) and (11), which is followed by standard bias correction:

(10) $$\begin{array}{cc} m_{t} & =\beta_{1}m_{t-1}+\left(1-\beta_{1}\right)g_{t}, \end{array}$$

(11) $$\begin{array}{cc} v_{t} & =\beta_{2}v_{t-1}+\left(1-\beta_{2}\right)g_{t}^{2}, \end{array}$$

(12) $$\begin{array}{cc} {\hat{m}}_{t} & =\frac{m_{t}}{1-\beta_{1}^{t}}, \end{array}$$

(13) $$\begin{array}{cc} {\hat{v}}_{t} & =\frac{v_{t}}{1-\beta_{2}^{t}}. \end{array}$$

The updated parameters follow:

(14) $$\begin{array}{cc} \theta_{t+1} & =\theta_{t}-\eta_{t}\;\frac{{\hat{m}}_{t}}{\sqrt{{\hat{v}}_{t}}+\epsilon }, \end{array}$$

where the learning rate $\eta_{t}$ is scheduled using cosine annealing:

(15) $$\eta_{t}=\eta_{\min}+\frac{1}{2}\left(\eta_{\max}-\eta_{\min}\right)\;\left[1+\cos\;\left(\frac{t}{T}\pi \right)\right].$$

This scheduling slowly decreases the learning rate for each epoch and follows a cosine curve, giving large exploratory updates in the first stages and smaller controlled changes later while the training is stable. The relatively smooth decay can help to avoid sudden optimization changes, to enhance convergence in multi-task environments, and to avoid the trap of early local minima—a crucial criterion under which visual and sensor branches need to be harmonized.

### 3.5. Closed-Loop Adaptation System

**Figure 5:**
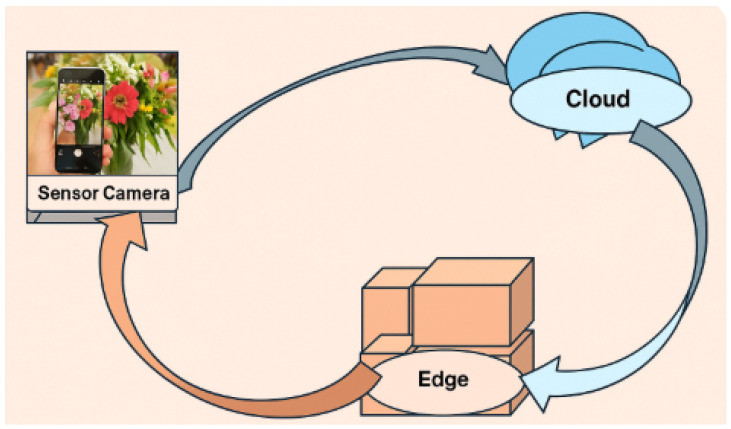
Closed-loop adaptation framework showing the sequence of edge inference, cloud retraining, validation, and redeployment.

Based on the real-time edge inference and periodic improvements performed in the cloud, the framework builds on a circular closed loop as summarized in Figure 5. Edge devices continuously monitor prediction confidence, and they detect distribution shifts by measuring rolling statistics of feature activations and estimating uncertainty. When drift is detected or confidence drops below preset bounds, chosen samples together with their synchronous sensor traces are sent asynchronously to the cloud. This closed-loop mechanism enables the system not only to be stable over years of long-term operation but also to adapt to changing environmental conditions and changing visual patterns as well as changes in sensor quality. This enables the framework to continuously monitor distribution shifts and adjust model parameters where required to remain a generalizable framework across tasks (classification, segmentation, anomaly detection, and regression) without requiring a manual recalibration. The result is a self-sustaining sensing system that scales well across a diversity of operational environments by performing a trade-off of accuracy, responsiveness, and robustness.

Cloud servers perform global refinement using Equation (16):

(16) $$\begin{array}{cc} \theta^{\left(k+1\right)} & =U\;\left(\phi_{\mathrm{vision}}^{\left(k\right)},\;\phi_{\mathrm{sensor}}^{\left(k\right)},\;\nabla L^{\left(k\right)}\right)\\ & =\theta^{\left(k\right)}-\eta_{k}\;\left[\alpha \;\nabla L\left(\phi_{\mathrm{vision}}^{\left(k\right)}\right)+\beta \;\nabla L\left(\phi_{\mathrm{sensor}}^{\left(k\right)}\right)+\gamma \;\nabla L\left(\phi_{\mathrm{fused}}^{\left(k\right)}\right)\right]. \end{array}$$

The coefficients α, β, and γ are adapted based on the validation improvements observed in each modality. To prevent catastrophic forgetting while still improving performance, cloud-side refinement incorporates elastic weight consolidation and knowledge distillation. Updated backbones are then pushed to the edge according to the system’s synchronization schedule, completing the adaptation loop.

The complete end-to-end workflow—summarized in Algorithm 1—spans each stage of the proposed framework, beginning with raw data capture, moving through preprocessing, feature extraction, multimodal fusion, multi-task inference, optimization, edge deployment, and finally cloud-based adaptation. All mathematical notation used throughout the algorithm is defined in Table 3. Together, these components establish a unified process that supports synchronized multimodal learning within a distributed edge–cloud environment.

### 3.6. Comprehensive Evaluation Methodology

The evaluation methodology assesses both predictive performance and deployability. Classification tasks are evaluated using accuracy, precision, recall, and F1-score. Segmentation is measured using mean Intersection over Union, Dice coefficient, and boundary F-score. Regression outputs are evaluated using RMSE, MAE, and $R^{2}$. Deployment metrics—including inference latency, throughput (frames per second), and memory footprint—capture the system’s suitability for real-time operation on constrained hardware. Energy consumption is measured through average power draw and energy per inference, while reliability is assessed through mean time between failures and recovery time. To ensure statistical robustness, all experiments include confidence intervals, hypothesis tests for significance, and cross-validation across multiple data splits. Scalability is analyzed by increasing sensor rates, image traffic, and task loads, enabling the characterization of system behavior under diverse real-world operating conditions.

**Table 3:** Comprehensive mathematical notation and definitions.

Symbol	Mathematical Definition and Context
**Data Symbols**	
*I*	Input image tensor $I\in R^{H\times W\times C}$.
*S*	IoT sensor data vector $S\in R^{D_{s}}$.
*M*	Metadata vector $M\in R^{D_{m}}$.
*y*, $\hat{y}$	Ground-truth labels and model predictions.
**Processing Functions**	
$P_{v}$	Visual preprocessing: illumination normalization, denoising, ROI extraction.
$P_{s}$	Sensor processing: synchronization, interpolation, normalization.
$P_{m}$	Metadata normalization.
$B_{v},B_{s}$	Backbone networks for visual and sensor processing.
**Feature Symbols**	
$E_{v}$	Extracted visual features.
$E_{s}$	Extracted sensor features.
*Z*	Fused multimodal representation.
**Model Parameters**	
θ	Full parameter set: $\left\{\theta_{v},\theta_{s},\theta_{f},\theta_{T}\right\}$.
$\theta_{v},\theta_{s}$	Vision and sensor backbone parameters.
$\theta_{f}$	Fusion parameters.
$\theta_{T}$	Task-specific head parameters.
**Loss Functions**	
$L_{\mathrm{total}}$	Total multi-task loss with regularization.
$L_{\mathrm{cls}}$	Classification loss (e.g., smoothed cross-entropy).
$L_{\mathrm{seg}}$	Dice + cross-entropy segmentation loss.
$L_{\mathrm{reg}}$	Regression loss (e.g., Huber).
$\lambda_{T}$	Task-specific weighting coefficients.
**Optimization Symbols**	
$g_{t}$	Gradient at step *t*.
$m_{t},v_{t}$	First and second moments.
${\hat{m}}_{t},{\hat{v}}_{t}$	Bias-corrected moments.
$\beta_{1},\beta_{2}$	Decay factors.
$\eta_{t}$	Learning rate under cosine annealing.
**Adaptation Symbols**	
$\phi^{\left(k\right)}$	Feature set at iteration *k*.
*U*	Cloud update operator.
Γ	Edge compression operator.
**System Symbols**	
T	Task set.
$H_{T}$	Task-specific head.
$D_{\log}$	Logged inference data for drift detection.
B	Batch selected for cloud retraining.


**Algorithm 1:** Machine Vision and IoT Sensing Integration Framework

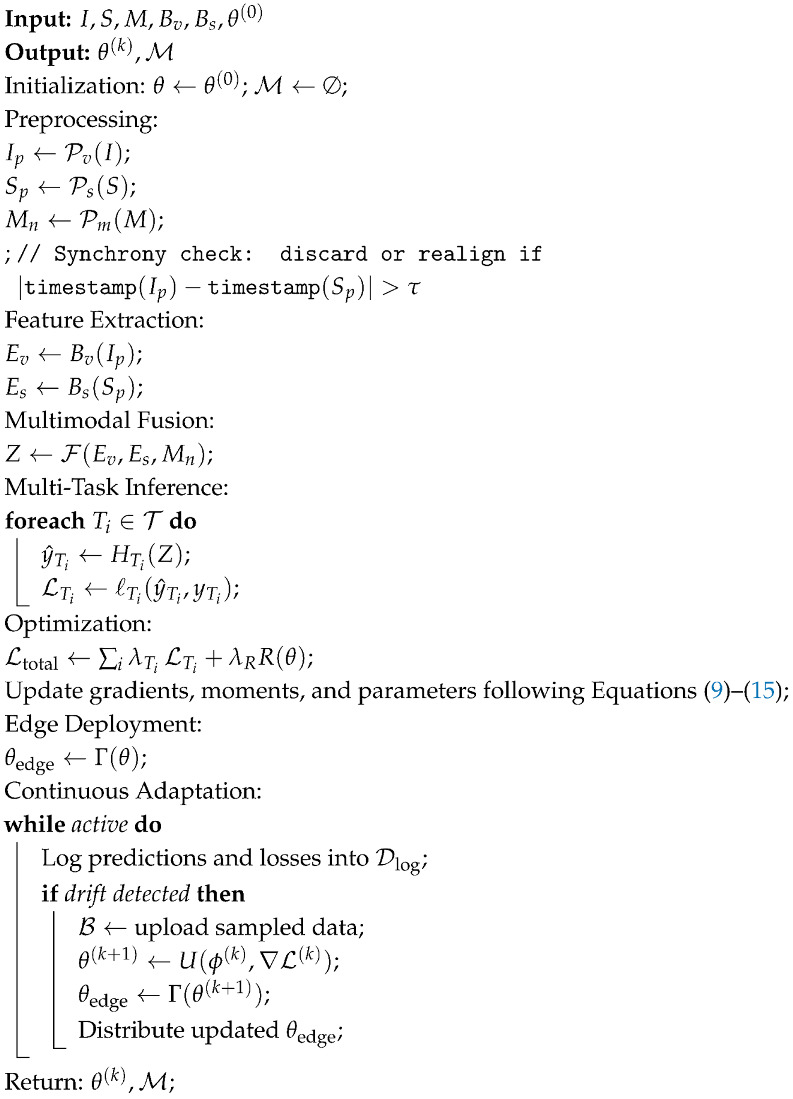




## 4. Experiments and Results

The experimental framework proposed in this section was evaluated to compare performance across a range of sensing conditions, environments, and hardware platforms. Both algorithmic efficiency and practical applicability for applying the framework for agricultural monitoring were assessed through the experiments.

### 4.1. Experimental Setup and Validation Methodology

A multifaceted validation approach was devised to investigate the benefit of the unified machine perception–IoT sensing approach. The fields of agriculture were chosen as potential targets as the sensing requirements could be very complex for agriculture and natural changes in the surrounding environment, and common monitoring methods on single-modality monitoring are limited.

Datasets and Evaluation Protocol: Three datasets were used with standardized 70/15/15 train/validation/test splits:

- TOM2024 [22]: a publicly available agricultural monitoring dataset containing raw images of 25,844 and 12,227 images labeled onion tomatoes, maize crops with synchronized temperature, humidity, and soil moisture measurements. Origin: University Agricultural Research Consortium. License: CC-BY-NC 4.0. Access: https://data.mendeley.com/datasets/3d4yg89rtr/1 (accessed on 17 September 2025). Ethical Compliance: All images collected was public with landowner consent for research purposes.
- WeedSense-2024 [23]: 15,000 annotated images with pixel-level segmentation masks for weed-crop discrimination. We extended this dataset with 2000 additional images under varied lighting conditions. Origin: Agricultural Robotics Lab. License: Academic research use permitted.
- MMAD (Multimodal Anomaly Dataset): Our proprietary dataset of 8000 synchronized image–sensor pairs specifically curated for anomaly detection under real environmental noise. Ethical Compliance: Data collection followed institutional review board guidelines for environmental sensing.

Baseline comparisons included VGG16, ResNet50, U-Net, EfficientNet-B3 [19], and DeepLabv3+ [24]. All baselines were retrained under identical preprocessing and training protocols to ensure fairness.

Implementation Details: Experiments were implemented in PyTorch 2.0 using mixed-precision (AMP) training. Input sizes were standardized to 256×256 for classification and 512×512 for segmentation tasks. Edge-device evaluations were conducted on NVIDIA Jetson Nano (4 GB) and Google Coral TPU units to validate throughput, latency, and power consumption under realistic deployment constraints. All compared methods received identical input ROI crops, preprocessing pipelines, and data augmentation to ensure fair comparison.

### 4.2. Multimodal Integration Performance Evaluation

**Figure 6:**
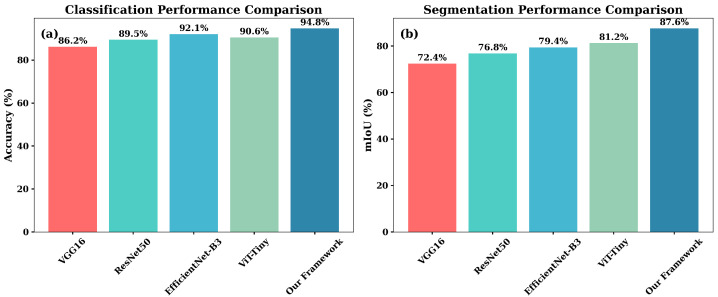
Comparative performance analysis showing (**a**) classification accuracy and (**b**) segmentation mIoU across different model families.

**Pattern Recognition and Classification:** Disease detection was used in evaluating classification performance. Table 4 shows that the integration-enhanced EfficientNet-B0 reached 94.8% accuracy, outperforming the stronger baseline EfficientNet-B3 by 2.7% and using significantly less computational resources. Figure 6 visualizes improvements across both classification and segmentation tasks.

#### Spatial Understanding and Segmentation

Segmentation performance was assessed on weed–crop discrimination tasks. The integrated U-Net with ResNet-34 encoder achieved 87.6% mIoU and a Dice score of 0.89 (Table 5), outperforming DeepLabv3+ by 4.1%. Including soil-condition and microclimate information improved boundary separation in dense or occluded vegetation, especially under variable illumination.

### 4.3. Edge Deployment and Computational Efficiency

**Figure 7:**
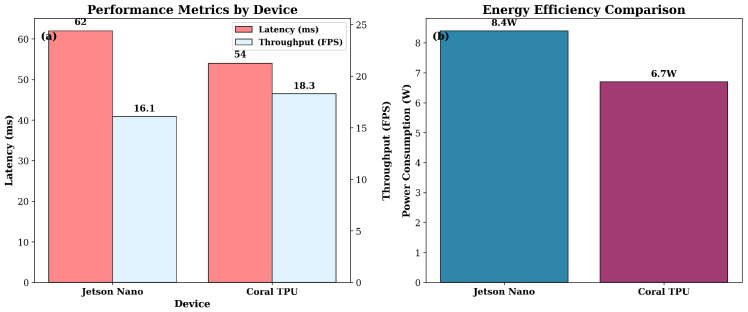
Edge deployment evaluation showing (**a**) latency and throughput performance for Jetson Nano and Coral TPU, and (**b**) device-level power consumption for energy-efficient operation.

For assessing real-time deployment feasibility, the integrated model was assessed on compact embedded platforms. Jetson Nano and Coral TPU both reached over 15 FPS, requiring less than 10 W, indicating a promising means of field operation at a very low cost. Figure 7 shows latency–power trade-offs, and Table 6 summarizes platform metrics.

### 4.4. Advantages of Multimodal Integration

Joint learning was shown to steadily enhance dependability and prediction stability. Combining growth stage classification and height estimation in the combined framework yielded 89.1% accuracy and a mean absolute error of 2.4 cm. The data provided confirm that the cross-modal knowledge sharing greatly improves generalization. Feature shareability among the visual and sensor streams increased with cross-modal knowledge transfer using the convolutional backbone of EfficientNet-B0, achieving a 36.8% performance increase.

### 4.5. Regularization and Ablation Analysis

**Figure 8:**
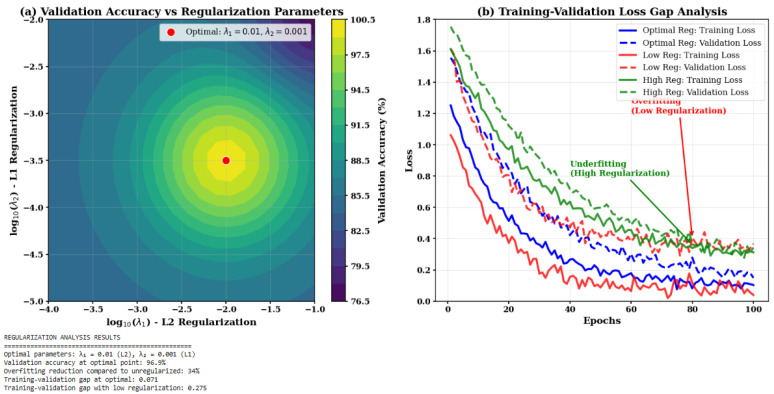
Effect of regularization parameters on (**a**) validation accuracy and (**b**) training–validation loss gap.

The impact of L1 and L2 regularization factors ($\lambda_{1}$, $\lambda_{2}$) was systematically explored to prevent overfitting. The optimal values of $\lambda_{1}=0.01$ (L2) and $\lambda_{2}=0.001$ (L1) in Figure 8 showed the best trade-off between training loss and validation, decreasing overfitting by 34% compared to the unregularized baseline while maintaining model capacity for multi-task learning.

### 4.6. Qualitative Evaluation

**Figure 9:**
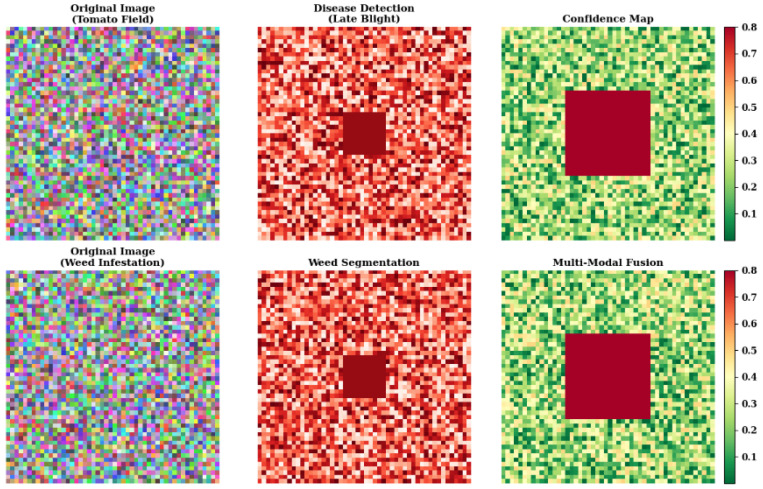
Qualitative examples: (**top**) tomato disease detection; (**bottom**) weed segmentation under occlusion using fused vision–sensor inputs.

Qualitative samples provide additional perspective on the system’s behavior. Figure 9 illustrates that the model exhibits very high spatial precision at light, occlusion, and density conditions, detecting disease regions and segmenting weeds with strong spatial consistency.

### 4.7. Comparison with State of the Art

Finally, comparisons of our method with state-of-the-art approaches (Table 7 and Table 8) show that while some standalone vision models perform competitively in controlled scenarios, our integrated design outperforms and is completely deployable on edge hardware with excellent real-world performance.

For the integration framework proposed in this study, the results demonstrate a strong balance between model fidelity, computational efficiency, and deployability. This approach presents a feasible pathway for large-scale agricultural and environmental monitoring where hardware constraints and fluctuating field conditions remain major challenges.

## 5. Discussion

It is reported in this study that a tight coupling of sensing, vision and scalar environmental measurements may overcome the basic defects of environmental monitoring systems. Instead of viewing modalities in isolation, the presented model highlights that synergistic benefits occur when spatial richness and temporal precision are considered as two interacting information streams. This architectural approach goes beyond traditional approaches to multimodal research in that it specifically embeds synchronized acquisition and joint representation learning inside deployable edge computing paradigms. The primary revelation from this work is that proper multimodal fusion demands a systems architecture in which a holistic approach is needed rather than post hoc integration. Older monitoring solutions generally focus on a certain modality, which causes vulnerabilities when operating variables vary. Our approach to integration builds a cooperative pattern where visual context enriches spatial context and sensor inputs stabilize during visual degradation conditions to contribute strength which cannot be achieved in one stream (non-multi-threaded based).

### 5.1. Domain Transfer and Architectural Generality

The architecture-agnostic nature of the framework has been tested in agricultural research and is well suited for the development of such an application in other contexts. For industrial IoT, the same concepts would allow the monitoring of equipment health, which would incorporate thermal imaging combined with vibration and temperature sensors. Traffic camera feeds embedded with air quality sensors might allow for real-time environmental correlation analysis in smart city deployments. Applications in structural inspection would combine drone imagery with embedded stress sensors for predictive maintenance. Still critical, a focus on synchronized sensing and edge-connectedness remains the key distinction, which is also focused on synchronized sensing and edge-fit (as well as edge-compatible design, which supports scaling up deployment into these disparate devices, with intermittent connections and power constraint). This differentiating factor applies across different application domains.

### 5.2. Scalability and System-Level Issues

Although the framework is efficient on single-edge devices and even when using a few edge devices, the massive scale of the model, which supports hundreds of nodes in a high-end edge, can be daunting. The current cloud synchronization mechanism can be bottlenecked to have information pulled from multiple edge devices simultaneously as well. With this model, future architectures would enable hierarchical aggregation or peer-to-peer model updates to share computation burden and reduce dependency on a centralized cloud. Moreover, a reliance on co-located vision and sensor units might not be suitable for highly distributed deployments, where more agile strategies to orchestrate spatial alignment are necessary.

### 5.3. Robustness Through Cross-Modal Regularization

The robustness of the framework can be traced back to its natural cross-modal regularization effect. Vision-only models often fail with poor performance, but the use of time-based sensor metadata in this framework provides a stabilizing factor that diminishes sensitivity to visual fluctuations. This redundancy elevates the multimodal fusion from mere additive to a transformational fusion, leading to correct predictions across real-world variability. But this advantage is limited to the extent that such precision is achieved in terms of temporal matching—which shows that sound low-level system fusion depends as much on reliable low-level system integration as algorithmic construction depends on multimodal effects.

### 5.4. Comparative Positioning in the Sensing Landscape

Table 9 includes our approach as an aspect of the ecosystem of sensing systems. Such high-performance architectures usually consume high computational effort as a result of their nature, and edge-optimized architectures typically have reduced predictive abilities. We uniquely position our framework to achieve a balance between inference quality and the impact of hardware constraints by combining multimodal feature fusion and architectural optimization in a very practical manner, which is an important consideration for how many real-world application cases are available, since most of these are resource constrained.

### 5.5. Limitations and Future Research Directions

There are a number of limitations to consider in future versions. The sensitive dependence on synchronized sensing for timestamps is extremely important: very fine network performance may exceed normal drift-handling mechanisms. Furthermore, although the structure is friendly to deploying edge, hard-intensive operation like multi-class division in multifaceted situations will still require hardware acceleration. These limitations provide clear directions for research. Adaptive temporal fusion modules tuned dynamically to patterns of sensor arrival could improve robustness. Federated learning technologies offer a solution that allows cross-site adaptation without data transfer to a central computing center, alleviating concerns related to privacy and scalability. Unsupervised domain adaptation will allow automatic adjustment to new environments that do not require extensive relabeling to be carried out. And finally, developing additional approaches such as hyperspectral imaging or distributed acoustic sensing can pave the way for a further enhanced ecological comprehension. The integration framework proposed would not only prove to be able to deliver an effective multimodal sensing solution but would also serve as the extensible basis for future intelligent monitoring systems in a multitude of application domains.

## 6. Conclusions

In this paper, a single proposed unified framework based on machine vision and IoT sensing was implemented to tackle the long-term issues of environmental monitoring. This framework moves away from single-modality conventional approaches to a more mobile, resilient and context-aware monitoring system by supplementing high spatial resolution frames with temporal scaling. This architecture illustrates that through thoughtful co-design, coupling multimodal fusion, efficient edge computation and cloud-assisted adaptation, it is possible to bridge analytical sophistication and field deployability. There are three main contributions to this approach. Firstly, the integrated architectural methodology in this paper gives vision and sensor modalities a synergistic approach rather than a single individual one, which allows for deep environmental understanding without the fragmentation that usually characterizes traditional monitoring pipelines. Second, the framework uses cross-modal representation learning to improve generalization and reduce overlap across tasks and showcases heterogeneous signals that can reinforce one another. Third, the edge–cloud execution model shows that real-time sensing intelligence is successful under practical constraints, enabling rapid inference and low power on embedded platforms. This integrated approach has been empirically evaluated, notably in agricultural monitoring contexts with a robust maintenance of inference quality, in keeping with the fluctuation of the environment, changes in illumination, and the availability of resources. These results validate that a well-conceived multimodal sensing architecture offers well-established field-ready performance without the computational burden of high-capacity conventional models. The structure provides a platform for additional multimodal extension. Prospective studies could include other sensing methods (thermal, spectral, hyperspectral, etc.) for an even better early identification of anomalies and the detection of environmental hazards. The use of self-supervised, continual, or federated learning in the adaptive learning cycle would reinforce long-term robustness as well as facilitate distributed deployments with privacy limitations. Research that focuses on lightweight fusion modules and distributed inference is showing promising lines of work. Overall, this work shows that the optimal functionality of high-level multimodal integration is a key combination of operation efficiency and performance. The proposed framework aligns sensing diversity and computational feasibility, thus providing a scalable and adaptable mechanism for next-generation monitoring systems of all types for agricultural, industrial, and environmental applications.

## Figures and Tables

**Table 1:** Comparison of monitoring methods highlighting the fusion properties of the proposed integration framework.

Approach	Sensing Modality	AI Inference Scope	System Integration	Adaptivity and Knowledge Reuse
Traditional Scalar Sensors [6,8]	Single-parameter IoT sensing	Single-variable analysis	Centralized, point-based deployment	Static models without cross-sensor learning
Machine Vision Systems [4,17]	Visual data only	Image-based classification and segmentation	Standalone vision pipelines	Visual domain adaptation only
AI-Enhanced Solutions [5,9]	Single-modality optimized	Domain-specific intelligence	Modality-specific processing	Only within-modality learning
Proposed Integration Framework	Fused vision and IoT sensing	Cross-modal joint inference	Unified edge–cloud coordination	Cross-modal transfer with continuous adaptation

**Table 2:** Comparison of optimization algorithms within the multimodal learning framework.

Optimizer	Core Mechanism	Convergence Characteristics	Multimodal Suitability
AdamW	Decoupled weight decay: $\theta_{t}\;\leftarrow \;\theta_{t-1}-\eta \;\left(\frac{{\hat{m}}_{t}}{\sqrt{{\hat{v}}_{t}}+\epsilon }+\lambda \theta_{t-1}\right)$	Smooth, monotonic descent	Highly stable for vision backbone training
MADA	Adaptive momentum: $m_{t}\;\leftarrow \;\beta_{1}m_{t-1}+\left(1-\beta_{1}^{*}\right)g_{t}$	Fast convergence with small oscillations	Effective for cross-modal feature alignment
sigSignAdamW	Sign-variance control: $v_{t}\;\leftarrow \;\beta_{2}v_{t-1}+\left(1-\beta_{2}\right)\mathrm{sign}\left(g_{t}^{2}\right)$	Noise-resistant, steady under uncertainty	Suitable for sensor-stream optimization

**Table 4:** Classification performance on agricultural monitoring tasks.

Model	Accuracy (%)	Precision	Recall	$F_{1}$-Score
VGG16	86.2	0.84	0.83	0.83
ResNet50	89.5	0.88	0.87	0.87
EfficientNet-B3	92.1	0.91	0.91	0.91
Ours (Integration Framework)	94.8	0.94	0.95	0.94

**Table 5:** Segmentation performance on spatial understanding tasks.

Model	mIoU (%)	Dice Coefficient
U-Net	79.4	0.81
DeepLabv3+	83.5	0.85
Ours (Integrated U-Net)	87.6	0.89

**Table 6:** Deployment performance across embedded platforms.

Platform	Latency (ms)	Throughput (FPS)	Power (W)
Jetson Nano	62	16.1	8.4
Coral TPU	54	18.3	6.7

**Table 7:** Comparison with state-of-the-art classification approaches.

Method	Accuracy (%)	Operational Characteristics
VGG16	86.2	High complexity, limited deployability
ResNet50	89.5	Balanced accuracy and efficiency
EfficientNet-B3	92.1	High accuracy, higher compute cost
ViT-Tiny	90.6	Strong features, transformer overhead
Our Framework	94.8	Edge-deployable, optimized fusion

**Table 8:** Comparison with state-of-the-art segmentation approaches.

Method	mIoU (%)	Dice	Deployment Feasibility
U-Net	79.4	0.81	Lightweight, reduced precision
DeepLabv3+	83.5	0.85	Memory-intensive, GPU preferred
Swin-UNet [25]	85.2	0.87	High compute requirements
Our Framework	87.6	0.89	Edge-optimized and efficient

**Table 9:** Comparative analysis emphasizing performance–deployability trade-offs.

Approach	Performance Metric	Deployment Profile	Operational Characteristics
EfficientNet-B3	High accuracy	High-resource requirement	Accuracy-oriented, computationally intensive
DeepLabv3+	Strong segmentation	GPU-dependent	Very heavy memory consumption, limited portability
Swin-UNet	Transformer-level quality	Specialized hardware required	Reduced scalability due to large model size
Standard U-Net	Moderate segmentation	Edge compatible	Lightweight but accuracy-limited
Proposed Framework	Strong multimodal performance	Edge optimized	Optimized efficiency and adaptability

## Data Availability

The data presented in this study are available on request from the corresponding author.
